# Associations between resting state brain activity and A_1_ adenosine receptor availability in the healthy brain: Effects of acute sleep deprivation

**DOI:** 10.3389/fnins.2023.1077597

**Published:** 2023-03-16

**Authors:** Changhong Li, Tina Kroll, Andreas Matusch, Daniel Aeschbach, Andreas Bauer, Eva-Maria Elmenhorst, David Elmenhorst

**Affiliations:** ^1^Institute of Neuroscience and Medicine (INM-2), Forschungszentrum Jülich, Jülich, Germany; ^2^Department of Neurophysiology, Institute of Zoology, RWTH Aachen University, Aachen, Germany; ^3^Department of Sleep and Human Factors Research, Institute of Aerospace Medicine, German Aerospace Center, Cologne, Germany; ^4^Division of Sleep Medicine, Harvard Medical School, Boston, MA, United States; ^5^Institute of Experimental Epileptology and Cognition Research, Faculty of Medicine, University of Bonn, Bonn, Germany; ^6^Institute for Occupational, Social and Environmental Medicine, Medical Faculty, RWTH Aachen University, Aachen, Germany; ^7^Division of Medical Psychology, Rheinische Friedrich-Wilhelms-University Bonn, Bonn, Germany; ^8^Multimodal Neuroimaging Group, Department of Nuclear Medicine, University Hospital Cologne, Cologne, Germany

**Keywords:** A_1_ adenosine receptor, acute sleep deprivation, resting-state fMRI, regional homogeneity, amplitude of low frequency fluctuations, degree centrality

## Abstract

**Introduction:**

Previous resting-state fMRI (Rs-fMRI) and positron emission tomography (PET) studies have shown that sleep deprivation (SD) affects both spontaneous brain activity and A_1_ adenosine receptor (A_1_AR) availability. Nevertheless, the hypothesis that the neuromodulatory adenosinergic system acts as regulator of the individual neuronal activity remains unexplored.

**Methods:**

Therefore, fourteen young men underwent Rs-fMRI, A_1_AR PET scans, and neuropsychological tests after 52 h of SD and after 14 h of recovery sleep.

**Results:**

Our findings suggested higher oscillations or regional homogeneity in multiple temporal and visual cortices, whereas decreased oscillations in cerebellum after sleep loss. At the same time, we found that connectivity strengths increased in sensorimotor areas and decreased in subcortical areas and cerebellum.

**Discussion:**

Moreover, negative correlations between A_1_AR availability and rs-fMRI metrics of BOLD activity in the left superior/middle temporal gyrus and left postcentral gyrus of the human brain provide new insights into the molecular basis of neuronal responses induced by high homeostatic sleep pressure.

## 1. Introduction

Sleep deprivation (SD) is a tool to study negative consequences of high homeostatic sleep pressure on brain and behavior. By using functional magnetic resonance imaging (fMRI), several meta-analysis studies (Ma et al., 2015; Yeo et al., 2015; Javaheripour et al., 2019; Saletin et al., 2019; Ning et al., 2022) revealed SD-induced changes in neuronal activity of brain regions, typically in the prefrontal cortex, thalamus, and intraparietal cortex regulating arousal, attention ability or emotional processing. Notably, several resting-state (Rs-)fMRI metrics, including amplitude of low-frequency fluctuations (ALFF) and its normalized version (fractional ALFF, fALFF), regional heterogeneity (ReHo), and degree centrality (DC), were applied to well characterize the differences in the regional properties of spontaneous brain activity (Zang et al., 2004, 2007; Zou et al., 2008; Zuo et al., 2012). In short, ALFF and fALFF are used to reflect the temporal fluctuations of low frequency oscillations (<0.1 Hz) across entire time series. ReHo is a voxel-based parameter for evaluating the similarity or synchronization of time series between a given voxel and its nearest neighbors. DC is an index of local functional connectivity (FC) strength and thus identifies the highest connected nodes by counting the number of direct connections to other nodes. Using these fMRI measures, we are capable to uncover voxel-based spectral and temporal characteristics of brain neuronal activity. For instance, prior Rs-fMRI studies (Gao et al., 2015; Wang et al., 2016; Chen L. et al., 2018) demonstrated reduced ALFF/fALFF values in the frontal and parietal cortex while increased values in the visual cortex and left sensorimotor cortex under the condition of sleep deprivation. Thus, the consistent changes across these fMRI properties should also be further investigated after acute sleep loss.

Generally, a neuronal response requires increased cellular energy metabolism that accelerates local cerebral blood flow or substrate delivery. With application of Positron Emission Tomography (PET), growing evidence (Wu et al., 1991; Thomas et al., 2000; Elmenhorst et al., 2007; Volkow et al., 2008, 2012; Qu et al., 2010; Holst et al., 2017) presented that acute SD remarkably upregulated A_1_ adenosine receptor (A_1_AR) availability, whereas glucose metabolism and dopamine D2/D3 neurons in human brain were reduced. Adenosine is a neuromodulator and directly linked to the energy metabolism as substrate of the breakdown of adenosine triphosphate. As a crucial mediator for promoting the homeostatic sleep drive, A_1_AR can be found in widespread brain regions (Bjorness and Greene, 2009; Huang et al., 2011). Elmenhorst et al. (2017) used [^18^F]-CPFPX PET and reported that sleep loss increased the A_1_AR availability in some cortical and subcortical brain regions, including the temporal cortex, striatum and insula. Meanwhile, neuronal alterations of these brain regions were also investigated by previous fMRI studies (Bell-McGinty et al., 2004; Venkatraman et al., 2007; Gujar et al., 2011; Krause et al., 2019). For instance, one earlier study (Krause et al., 2019) confirmed that sleep loss extremely amplified pain reactivity within primary somatosensory cortex but reduced pain reactivity in higher-order evaluation and decision-making regions of the striatum and insula. For those above-mentioned overlapped regions, no neuroimaging studies investigated so far whether changes in their neuronal activity are associated with corresponding changes in A_1_AR availability. Therefore, we aimed to evaluate the contribution of A_1_AR in the specific regional properties of neural activities of some key brain regions by combing these two independent modalities.

Hence, our present study intends to explore the alterations in common Rs-fMRI metrics after 52 h of SD (SD52) and after 14 h of recovery sleep (RS14), as well as whether potential changes in these Rs-fMRI metrics are associated with changes in A_1_AR availability and behavioral performance. To this end, we first conducted voxel-based statistical comparisons of ALFF/fALFF, ReHo, and DC after SD52 in comparison with RS14. Then, we correlated the differences (SD52 - RS14) in A_1_AR availability with the differences in Rs-fMRI metrics of those significant clusters. Lastly, we tested the correlations between the differences (SD52 - RS14) in the above neuroimaging parameters and corresponding neuropsychological performance.

## 2. Materials and methods

### 2.1. Participants and study protocol

**FIGURE 1:**
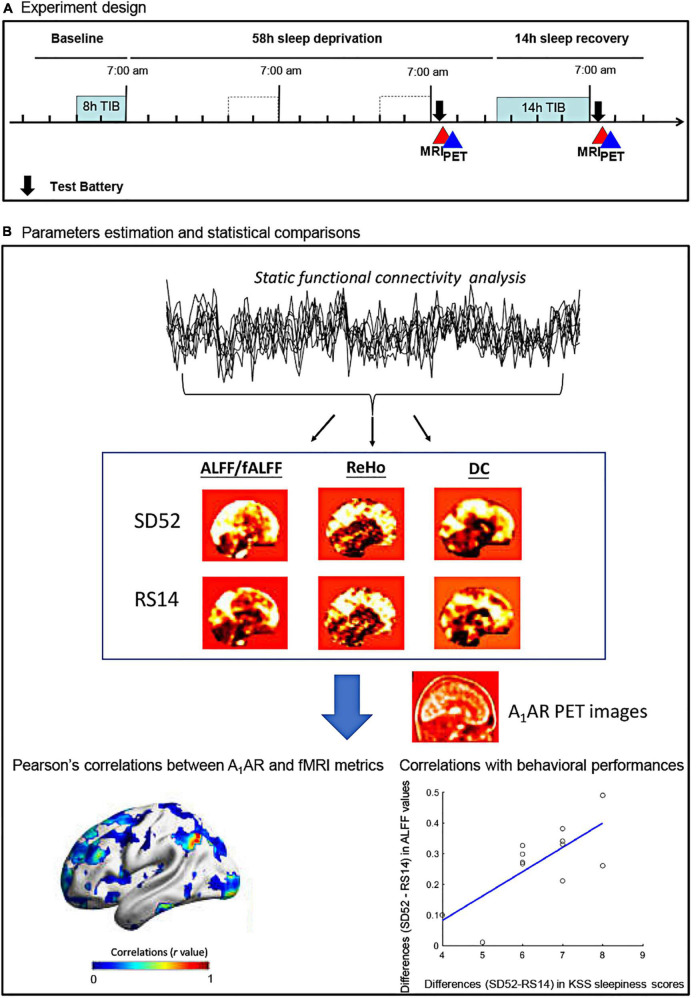
Flowchart of the study protocol for the 52 h of sleep deprivation. ALFF, amplitude of low frequency fluctuations; fALFF, fractional amplitude of low frequency fluctuations; ReHo, regional heterogeneity; DC, degree centrality; KSS, Karolinska Sleepiness Scale; SD52, 52 h of sleep deprivation; RS14, 14 h of recovery sleep; A_1_AR, A_1_ adenosine receptor; TIB, time in bed.

Fourteen young males (age: 28.21 ± 5.21 years, mean body mass index: 24.39 ± 3.58) were recruited. All participants were interviewed to ensure that they did not have any neurological or psychiatric diseases prior to the experiments. Each subject underwent PET, Rs-fMRI scans, and neuropsychological tests twice, at SD52 and at RS14 conditions (Figure 1A). Neuropsychological examinations included a 3-min version of psychomotor vigilance test (PVT), spatial 3-back working memory task, and sleepiness rating scale (Karolinska Sleepiness Scale, KSS). PVT-speed, PVT-lapses, 3-back hits, 3-back reaction time, as well as KSS sleepiness scores were derived. Details on inclusions of all participants, procedures of MRI/PET scans, and neuropsychological testing have been documented in the Supplementary material and our earlier publications (Elmenhorst et al., 2017; Li et al., 2020). This study was approved by the Ethics Committee of the Medical Faculty of the University of Düsseldorf and informed consent was obtained from all participants.

### 2.2. Rs-fMRI and [^18^F]-CPFPX PET acquisitions

A 3T Siemens MAGNETOM Trio MRI scanner (Erlangen, Germany) with a 32-channel head coil was used to obtain MRI datasets. Subjects were instructed to keep the eyes open and to focus on a “+” that was presented on a screen positioned at the end of the gantry. By using a gradient-echo echo planar imaging sequence, we acquired Rs-fMRI datasets with following parameters: Time of Repetition (TR) = 2.2 s, Echo Time (TE) = 30 ms, Flip angle = 90°, matrix size = 64 × 64, 36 slices, slice thickness = 3.1 mm, voxel size = 3.1 × 3.1 mm^2^, 146 volumes in total. Meanwhile, we conducted a 3D magnetization-prepared rapid acquisition gradient echo (MPRAGE) anatomical scans (TR = 2.25 s, TE = 3.03 ms, matrix size = 256 × 256, 176 slices, voxel size = 1 × 1 mm^2^, slice thickness = 2.25 mm, flip angle = 90°).

For the collection of [^18^F]-CPFPX PET datasets, we used a Siemens ECAT EXACT HR+ scanner (Siemens-CTI). The radiotracer was injected as a bolus followed by a constant infusion with a *K*_*bol*_ value of 55 min. Scan duration was 100 min. Arterialized venous blood sampling was taken at minute timepoints 1, 5, and 10, and every 10 min subsequently.

### 2.3. Rs-fMRI dataset preprocessing and analysis

Figure 1B showed the detailed flowchart of our study. All datasets were preprocessed using DPABI_V3.1^1^ and SPM12 (7219).^2^ In details, first 5 volumes were discarded to ensure that the subject has adapted to the scanner and to remove potential head movements. Secondly, we performed slice-timing and head motion correction. During this step, no subjects were excluded because inclusion criteria were fulfilled: head transitions < 3 mm, rotations < 3° or mean Framewise Displacement (FD) value < 0.35 mm. As a result, mean FD values of two conditions were estimated as follows: SD52, 0.16 ± 0.06 mm; RS14, 0.15 ± 0.07 mm. Thirdly, Friston 24-motion parameters and signals from cerebrospinal fluid and white matter were regressed out. We then normalized the preprocessed Rs-fMRI images to the standard MNI template space using the DARTEL algorithm and removed a linear trend from the time series of each subject. Lastly, we performed band-pass filtering (0.01 ∼ 0.08 Hz). No global signal regression was applied. To generate the parametric maps of ALFF/fALFF and DC, we smoothed the normalized Rs-fMRI images with 8 mm^3^ Full Width at Half Maximum (FWHM). Given some specific frequency bands of spontaneous brain activity were thought to have distinct properties and physiological functions (Buzsaki and Draguhn, 2004; Zuo et al., 2010; Han et al., 2011), we additionally applied Slow-5 (0.01–0.027 Hz) and Slow-4 (0.027–0.073 Hz) to replicate our findings of ALFF/fALFF analysis.

In details, the power spectrum in three frequency ranges of 0.01–0.08 Hz, Slow-5 and Slow-4 range were separately computed at whole-brain voxel-wise level. We then *z*-transformed the ALFF values before the statistical analyses. The fALFF were conducted by their rations of ALFF values relative to full frequency range (0–0.25 Hz).

For other parameters, we exhibited the equations:


(1) $$D\,C\,\left(i\right)=\int_{j=1}^{N}{\text{a}}_{i\,j}$$

In a weighted graph, the element *a*_*ij*_ indicates the connection or edge from a specific node *i* to its connecting node *j*.


(2) $$W=\frac{\sum {\left(R_{i}\right)}^{2}-n\,{\left(\bar{R}\right)}^{2}}{\frac{1}{12}\,K^{2}\,\left(n^{3}-n\right)}$$

where *W* is the Kendall’s coefficient of concordance among given voxels, ranged from 0 to 1; *R*i is the sum rank of the i*^th^* time point; where $\bar{R}$ is the mean of the (n+1)K/2; *K* is the number of time series within a measured cluster (*K* = 27 in our study).

### 2.4. PET preprocessing and calculations

We performed realignment, coregistration, segmentation, and normalization of the PET dataset to standard MNI152 space with PMOD (v3.305, PMOD Group). The total distribution volume *V*_*T*_ in the equilibrium (between 50 and 100 min) is calculated as *V*_*T*_ = TAC/C_*p*_, with TAC represents the tissue activity concentration of the radioligand and *C*_*p*_ indicates plasma activity of parent compound (Elmenhorst et al., 2007). After identifying the significant clusters of local Rs-fMRI metrics, we extracted their corresponding mean A_1_AR distribution volumes for each region. During the voxelwise correlations analysis, we smoothed the PET dataset with 8 mm^3^ FWHM to increase the spatial coherence.

### 2.5. Statistical comparisons

Considering the small sample size, we applied a Permutation Analysis of Linear Models approach (PALM, 5000 permutations) (Winkler et al., 2014) to perform two-sample paired *t*-test across all local Rs-fMRI metrics. It should be noted that a permutation test with Threshold-Free Cluster Enhancement (TFCE) approach reached the best balance between false wise error rate (FWER) and test–retest reliability in an earlier study (Chen X. et al., 2018). Hence, the significant threshold was determined at one-tailed FWER < 0.05 with minimum cluster size > 10 voxels, which ran in DPABI_V3.1 toolbox. During the statistical comparisons, mean FD values were carried out as variance of no interest.

### 2.6. Voxelwise and regional-based correlations analysis

Owing to the failures of acquiring one of the two PET images in two participants, we only retained 12 participants for further correlation analysis. We extracted the mean A_1_AR values for those significant clusters in the fMRI properties and calculated the differences (SD52 - RS14) for each subject. Using a combination of PALM (5000 permutations) and TFCE approaches, we computed the whole brain voxelwise Pearson correlation between the differences (SD52 - RS14) in A_1_AR distribution volumes and the differences in each of Rs-fMRI metrics. Statistical threshold was determined at one-tailed FWER < 0.05. For the region of interest (ROI)-based analysis, we used Pearson correlation to investigate the associations between differences (SD52 - RS14) in A_1_AR distribution volumes of those significant clusters and the differences in corresponding Rs-fMRI metrics. Finally, we applied Pearson correlations to separately examine the relationships of the differences (SD52 - RS14) in local Rs-fMRI metrics and A_1_AR distribution volumes with the differences in neuropsychological tests (KSS sleepiness scores, PVT-lapses, PVT-speed, and 3-back hits).

## 3. Results

### 3.1. Sleep deprivation-induced alterations in local Rs-fMRI metrics

**FIGURE 2:**
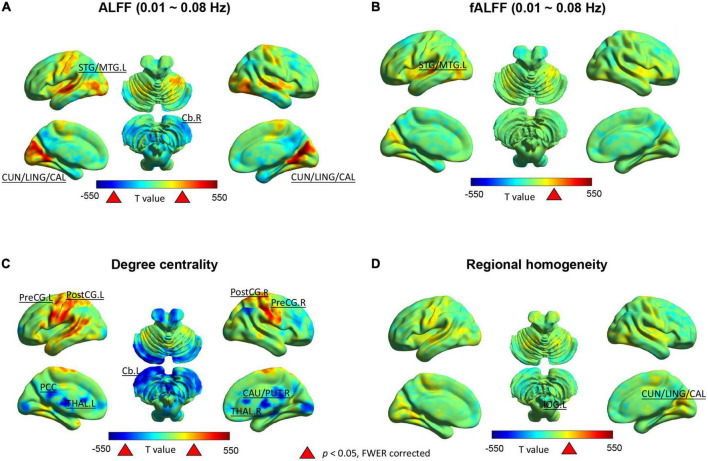
Between-group differences (52 h of sleep deprivation–14 h of recovery sleep) in the Rs-fMRI metrics for 14 healthy participants. Positive *t*-value indicates the significant increases and negative *t*-value represent decreases after 52 h of sleep deprivation. ALFF, amplitude of low frequency fluctuations; fALFF, fractional amplitude of low frequency fluctuations; L, left hemisphere; R, right hemisphere; STG/MTG, superior/middle temporal gyrus; CUN/LING/CAL, cuneus/lingual/calcarine; Cb, cerebellum; PostCG, postcentral gyrus; PreCG, precentral gyrus; PCC, posterior cingulate cortex; CAU/PUT, caudate/putamen; THAL, thalamus.

In the typical frequency band (0.01 ∼ 0.08 Hz), we observed significantly higher ALFF values in the bilateral cuneus/lingual/calcarine and left superior/middle temporal gyrus, but lower values in the right cerebellum after SD52 compared to RS14 (Figure 2A). We only observed significantly increased fALFF values in the left superior/middle temporal gyrus (Figure 2B). As illustrated in Figure 2C, acute sleep loss significantly increased DC values in the bilateral precentral gyrus and postcentral gyrus, whereas DC values were significantly decreased in the thalamus, posterior cingulate cortex, right putamen/caudate, and left cerebellum. Regarding the ReHo maps, the values of the bilateral cuneus/lingual/calcarine were significantly higher after SD52 compared to RS14 (Figure 2D).

In the Slow-5 frequency band, only the ALFF values in the right cerebellum were significantly lower (Supplementary Figure 1A). However, in the Slow-4 band significantly increased ALFF was found in the bilateral superior/middle temporal gyrus, bilateral cuneus/lingual/calcarine, left inferior occipital gyrus, and right fusiform gyrus (Supplementary Figure 1B). For the fALFF maps in the typical frequency band and Slow-4, we found significantly higher values in the left superior/middle temporal gyrus after SD52 (Supplementray Figure 1C).

### 3.2. Correlation analysis between A_1_AR availability and Rs-fMRI metrics

**FIGURE 3:**
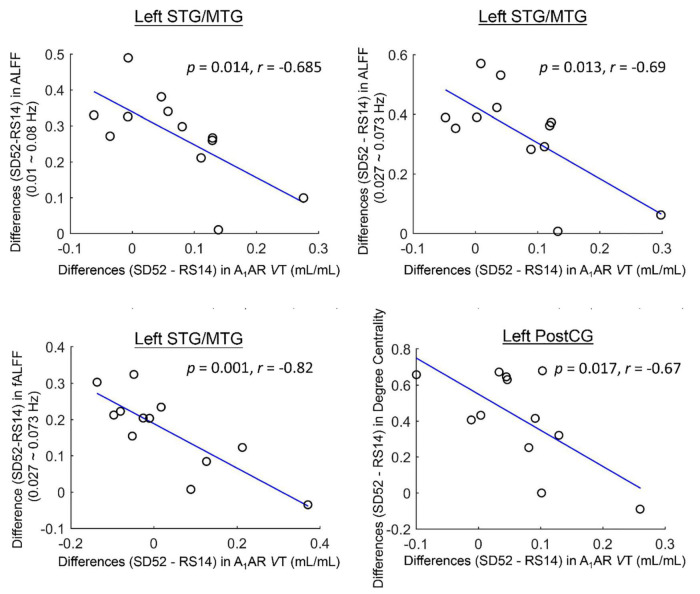
Cluster-based associations between the differences (52 h of sleep deprivation–14 h of recovery sleep) in A_1_AR distribution volumes and Rs-fMRI metrics for 12 healthy participants. STG/MTG, superior/middle temporal gyrus; PostCG, postcentral gyrus; ALFF, amplitude of low frequency fluctuations; fALFF, fractional amplitude of low frequency fluctuations; VT, distribution volume.

For the ROI-based comparisons, the differences in A_1_AR distribution volumes within the left superior/middle temporal gyrus correlated negatively with the differences in mean ALFF values of the typical frequency band and Slow-4, as well as mean fALFF values of Slow-4 (*r* = −0.685, *p* = 0.014; *r* = −0.69, *p* = 0.013; *r* = −0.82, *p* = 0.001; Figure 3). The differences in A_1_AR distribution volumes of left postcentral gyrus correlated negatively with the differences in DC values (*r* = −0.67, *p* = 0.017; Figure 3).

For voxelwise comparisons with FWER correction, we did not find any significant correlations between the differences (SD52 - RS14) in A_1_AR distribution volumes and the differences in Rs-fMRI metrics (Supplementary Figure 2).

### 3.3. Correlations with neuropsychological tests

We detected that the differences (SD52 - RS14) in mean ALFF values (0.01 ∼ 0.08 Hz) and DC values of the PCC area were negatively correlated with the differences of PVT-speed (Supplementary Table 1, *r* = −0.67, *p* = 0.009; *r* = −0.75, *p* = 0.002). The differences (SD52 - RS14) in mean ALFF values of the left superior/middle temporal gyrus, mean fALFF values of the left middle temporal gyrus, and DC values of the left postcentral gyrus correlated positively with the differences in KSS sleepiness scores (*r* = 0.63, *p* = 0.016; *r* = 0.54, *p* = 0.047; *r* = 0.71, *p* = 0.004). However, the differences in DC values of the posterior cingulate cortex and left cerebellum correlated negatively with the differences in KSS sleepiness scores (*r* = 0.71, *p* = 0.004; *r* = −0.74, *p* = 0.002). The differences of mean ALFF values of the typical frequency and Slow-4 of the cuneus/lingual/calcarine area were negatively correlated with the differences of 3-back rection times (*r* = −0.54, *p* = 0.045; *r* = −0.54, *p* = 0.047). Differences in A_1_AR availability in several of these identified brain regions correlated with the differences in PVT-lapses, 3-back hits, and KSS sleepiness scores (please refer to Supplementary Table 2 for more details).

## 4. Discussion

This study systematically performed whole brain voxel-wise comparisons across local Rs-fMRI metrics of spontaneous brain activity (ALFF/fALFF, ReHo, and DC) comparing SD52 to RS14. After acute sleep loss, we identified both significant higher amplitude of low-frequency fluctuations (ALFF) and ReHo values in cuneus/lingual/calcarine. Meanwhile, ALFF values increased significantly in the superior/middle temporal gyrus, whereas they decreased in right cerebellum. Our findings also showed significantly higher degree centrality in bilateral precentral gyrus and postcentral gyrus, but decrease in thalamus, posterior cingulate cortex, right caudate/putamen, and left cerebellum. Furthermore, our correlation analysis suggested an increase in the differences (SD52 - RS14) of A_1_AR availability were correlated with a decrease in the differences of mean ALFF/fALFF in left/middle temporal gyrus and degree centrality in left postcentral gyrus. An increase in the differences (SD52 – RS14) of local Rs-fMRI metrics within several brain regions significantly correlated with an impairment in PVT-speed and high feelings of subjective sleepiness, whereas lower A_1_AR availability of most brain regions were associated with larger impairments in cognitive performances (PVT-lapses and 3-back hits).

Of these findings, the most prominent one is that high homeostatic sleep pressure synchronized amplitude of low frequency fluctuations of blood-oxygen-level-dependent (BOLD) activity in primary visual cortex, which was reflected by the increased ALFF/fALFF and ReHo in bilateral cuneus/lingual/calcarine after sleep loss. Similar results of higher ALFF values after 24 or 72 h of sleep loss have already been reported in two Rs-fMRI studies (Gao et al., 2015; Wang et al., 2016). Meanwhile, we also noticed the remarkably decreased degree centrality in this region, which is consistent with earlier findings (Farahani et al., 2019; Xu et al., 2021). After reviewing the behavioral consequences in 21 studies, one earlier study (Waters et al., 2018) concluded that sleep restriction mainly deteriorated the visual domains (90% of these studies) in healthy participants, such as metamorphopsia, illusions, and hallucinations. Additionally, one fluorodeoxyglucose (FDG)-PET study (Thomas et al., 2000) identified a decreased cerebral metabolic rate of glucose uptake within this brain area. In the frequency band of Slow-4, our results extended the sleep-loss related changes to the right fusiform gyrus and left inferior occipital gyrus, which were discovered in other fMRI studies as well (Gao et al., 2015; Chen L. et al., 2018). Though the nature and their pathological functions of low frequency bands were not fully identified yet, some brain regions are more sensitive to one of these two different neighboring frequency bands in the neuroimaging studies of human brain (Hutchison et al., 2004; Han et al., 2011; Li et al., 2017). Combined with their correlations with outcomes of PVT, we propose that the upregulated power spectrum of BOLD signals in these visual areas primarily delayed the response speed in the attentional task. Meanwhile, our results revealed increased ALFF/fALFF values in bilateral superior/middle temporal gyrus but attenuated ALFF values in right cerebellum after sleep loss. Using single-neuron recordings in the human neurosurgical patients, one study (Nir et al., 2017) that reported that sleep deprivation induced prolonged and weakened spiking responses of individual neurons in middle temporal gyrus prior to cognitive lapses during a face/non-face categorization PVT. Lastly, the reduced power spectrum of BOLD activity within the right cerebellum, which was in line with a prior study (Chen L. et al., 2018), may reflect the accelerated oxygen consumption in order to sustain movement, emotional and cognitive functions.

Using the index of degree centrality, we identified hyperconnectivity within the sensorimotor cortex (PreCG and PostCG), but reduced strengths in some hub regions of the default-mode network (PCC) and subcortical network (thalamus and CAU/PUT), as well as left cerebellum. After sleep deprivation, earlier study (Gorgoni et al., 2014) observed enhanced excitability with an amplitude increase of somatosensory evoked potentials. Moreover, they found that voltage changes correlated with post-SD fluctuations of subjects’ sleepiness. Meanwhile, the decrease of thalamocortical connectivity after sleep loss has been shown to be critical for attention and arousal regulation in previous studies (Portas et al., 1998; Chee et al., 2008; Tomasi et al., 2009; Shao et al., 2013; Liu et al., 2018). More specifically, thalamic activity was decreasing with lower arousal level during resting-state but conversely elevated when the subjects were required to perform an attention task. Notably, reductions of FC strengths between thalamus and cortical areas were reported under the conditions of coma, general anesthesia, and non-rapid eye movement sleep (Kaufmann et al., 2006; Akeju et al., 2014; Picchioni et al., 2014; Hannawi et al., 2015). Therefore, these findings suggest that the thalamus acts as “control switch” to regulate human brain consciousness states. In the end, the FC strengths within the posterior cingulate cortex, right PUT/CAU of basal ganglia and left cerebellum were declined because of post-SD sleepiness, which was consistent with previous findings (Lazarus et al., 2013; Tomasi et al., 2016). To sum up, higher connectivity strengths within sensorimotor cortex but a decrease in strength in the default mode and subcortical network after sleep loss were closely associated with attentional deficiencies and sleepiness.

Most importantly, our findings highlight the negative associations between A_1_AR distribution volumes and Rs-fMRI metrics in mean ALFF/fALFF values of left superior/middle temporal gyrus and degree centrality of left postcentral gyrus. To our knowledge, this is the first study to investigate their associations by combining PET and Rs-fMRI datasets whereas prior work was limited to separate investigations of the brain’s A_1_AR availability and BOLD activity (Gao et al., 2015; Elmenhorst et al., 2017). Noticeably, the A_1_AR availability of superior/middle temporal gyrus were elevated in terms of some antidepressant therapies in the patients with major depression disorders, such as electroconvulsive therapy, deep brain stimulation, or transcranial magnetic stimulation (Sakagami et al., 2005; Bekar et al., 2008; Kato, 2009; Hamani et al., 2010). These findings may further indicate that the effectiveness of sleep restriction on emotional regulation of depressive patients might be accomplished by the accumulation of A_1_ adenosine receptors density, which was correlated to slower oscillations of BOLD activity. With respect to the left postcentral gyrus, the decreases in A_1_AR availability are associated with reduced FC strengths between sensorimotor cortex and other brain regions. Additionally, significant associations of A_1_AR availability with PVT-lapses and 3-back hits indicate the high A_1_AR bindings within these brain areas might be the molecular mechanism of spatial neglects during sustained attention, which was discussed in a previous publication (Elmenhorst et al., 2017).

Nevertheless, the sample size (*N* = 14) of our study is relatively small and hence restricted the statistical power for detecting between-conditions differences. To solve this issue, we performed Permutation Analysis Linear Model with 5000 repetitions to produce the statistical inferences. Secondly, we should keep in mind that the neuronal or metabolic activity of the human brain after recovery sleep may not be fully restored to rested baseline level (Wu et al., 2006; Chai et al., 2020). Lastly, several PET studies (Wu et al., 1991; Chuah and Chee, 2008; Volkow et al., 2008, 2012) reported that acute sleep deprivation reduced glucose uptake, D2/D3 neurons, but increased cholinergic neurons in some brain areas. Therefore, the complex relationships among the BOLD activity, different types of neurotransmitters and behavioral outcomes should be investigated further.

## 5. Conclusion

In our current study, for the first time, negative correlations between A_1_AR availability and BOLD activity in the left superior/middle temporal gyrus and left postcentral gyrus of the human brain provide new insights into the molecular basis of neuronal responses induced by high homeostatic sleep pressure.

## Data availability statement

The raw data supporting the conclusions of this article will be made available by the authors, without undue reservation.

## Ethics statement

The studies involving human participants were reviewed and approved by the Ethics Committee of the Medical Faculty of the University of Düsseldorf. The patients/participants provided their written informed consent to participate in this study.

## Author contributions

CL conceived the presented idea, performed the computations, and wrote the manuscript. DE and E-ME designed and carried out the experiments and revised the manuscript. TK, AM, DA, and AB discussed the results and contributed to the final version of manuscript. All authors contributed to the article and approved the submitted version.

## Footnotes

1
http://rfmri.org/dpabi

2
http://www.fil.ion.ucl.ac.uk/spm/software/spm12/
