# Supplementary material for: Associations between resting state brain activity and A1 adenosine receptor availability in the healthy brain: Effects of acute sleep deprivation
Source: Front Neurosci. 2023 Mar 16;17:1077597. doi: 10.3389/fnins.2023.1077597 (PMC10062390; doi:10.3389/fnins.2023.1077597)
Supplement: Supplementary file 1 [file Data_Sheet_1.docx]

Supplementary Material

# Supplementary method and materials

*Participants and study protocol*

All the participants were recruited through the Internet and local newspaper. They are non-smokers and non-alcoholics. One week before arriving the lab, these subjects were instructed to keep a regular sleep schedule (time in bed: 23:00 pm to 7:00 am). They were not allowed to consume caffeine (confirmed by plasma caffeine-determination) and wear a wrist-actigraphy at 4, and 3 days before the experiment, respectively. When they arrived the lab, participants were kept awake for consecutive 52 hours. Finally, they had a 14 hours recovery sleep opportunity (21:00-7:00 hours or 22:00-8:00 hours). The participants were monitored by our research staff all the time. Light level was set at ~650 lx. Each subject underwent neuropsychological tests and [^18^F]-CPFPX PET at the same time of day in the late morning, two hours later, Rs-fMRI scans were acquired accordingly (Fig. 1). The neuropsychological tests included 3-min version of psychomotor vigilance test (PVT) PVT-speed, spatial 3-back working memory task, and PVT-lapses, and sleepiness rating scale (Karolinska Sleepiness Scale, KSS). In detail, PVT measures the subjects’ sustained attention ability, which required the subject response the lighting up of the battery lamp on a portable, handheld computer. Lapses were calculated as responses longer than a limited reaction time (500 ms). Meanwhile, a conventional 3-back spatial working memory task is designed to estimate working memory ability in our study. 3-back hit is the reaction to the specific stimulus that were shown the three earlier. Karolinska Sleepiness Scale (KSS) is a 10-points self-report questionnaire that can evaluate the subjunctive’s sleepiness level, which is sensitive to the fluctuated sleepiness and reflects the psycho-physical state level in the last 10 min.

# Supplementary Figures and Tables

**
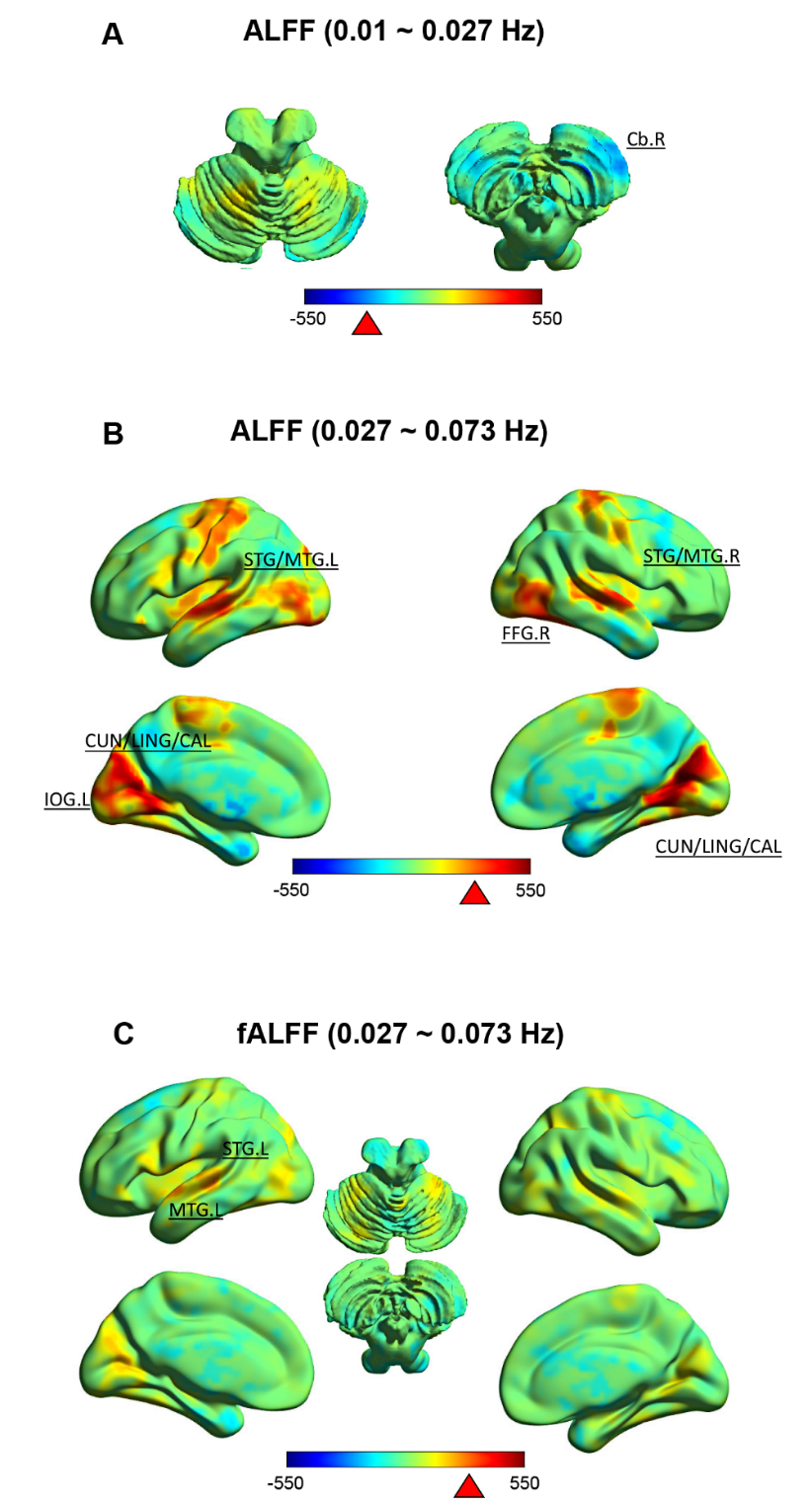
**

**Supplementary Figure 1.** Significant differences in ALFF and fALFF values (Slow-5 and Slow-4 bands) between 52 hours of sleep deprivation and 14 hours of recovery sleep.

Abbreviations: ALFF, amplitude of low frequency fluctuations; fALFF, fractional amplitude of low frequency fluctuations; L, left hemisphere; R, right hemisphere; STG/MTG, superior/middle temporal gyrus; CUN/LING/CAL, cuneus/lingual/calcarine; Cb, cerebellum; IOG, inferior occipital gyrus; FFG, fusiform gyrus.

**
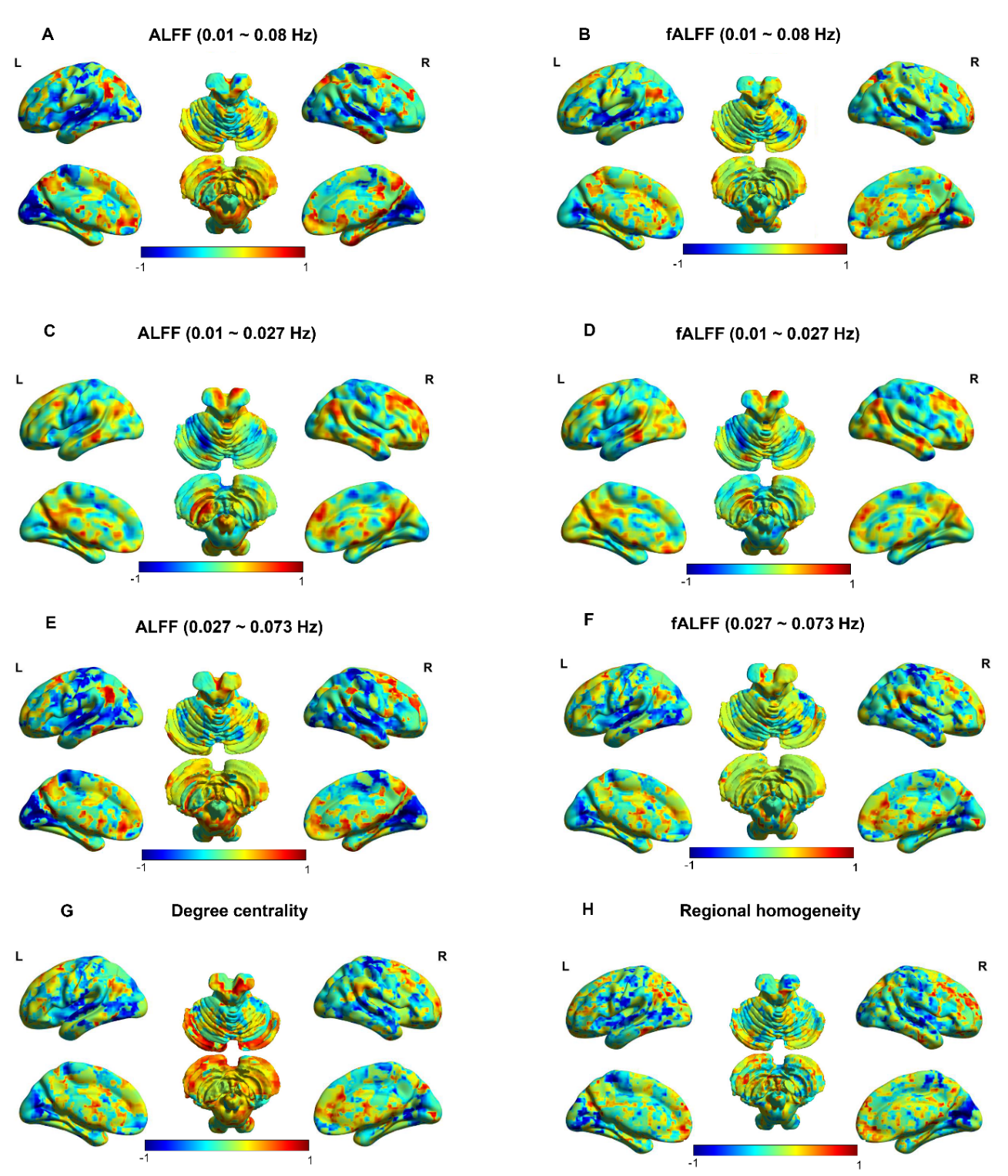
**

**Supplementary Figure 2.** Whole brain voxelwise associations between the differences (SD52 - RS14) in A1AR distribution volumes and Rs-fMRI metrics.

Abbreviations: ALFF, amplitude of low frequency fluctuations; fALFF, fractional amplitude of low frequency fluctuations; L, left hemisphere; R, right hemisphere.

**Supplementary Table 1** Correlations between the difference (52 hours of sleep deprivation - 14 hours of recovery sleep) in Rs-fMRI metrics and neuropsychological tests for 14 healthy participants.

| Rs-fMRI metrics | | PVT-speed | PVT-lapses | 3-back hits | KSS sleepiness scores | 3-back reaction time |
| --- | --- | --- | --- | --- | --- | --- |
| ALFF (0.01 ~ 0.08Hz) | STG/MTG.L | *r* = -0.09, *p* = 0.75 | *r* = 0.19, *p* = 0.504 | *r* = 0.08, *p* = 0.789 | ***r* = 0.63, *p* = 0.016** | *r* = 0.15, *p* = 0.605 |
|  | CUN/LING/CAL | ***r* = -0.67, *p* = 0.009** | *r* = 0.32, *p* = 0.266 | *r* = -0.09, *p* = 0.766 | *r* = 0.42, *p* = 0.131 | ***r* = 0.54, *p* = 0.045** |
|  | Cb.R | *r* = 0.23, *p* = 0.42 | *r* = -0.27, *p* = 0.351 | *r* = 0.44, *p* = 0.115 | *r* = -0.48, *p* = 0.079 | *r* = -0.33, *p* = 0.250 |
| fALFF (0.01 ~ 0.08 Hz) | STG/MTG.L | *r* = -0.37, *p* = 0.196 | *r* = 0.06, *p* = 0.843 | *r* = -0.15, *p* = 0.599 | ***r* = 0.58, *p* = 0.032** | *r* = 0.19, *p* = 0.506 |
| ALFF (0.01 ~ 0.027 Hz) | Cb.R | *r* = -0.19, *p* = 0.516 | *r* = -0.10, *p* = 0.732 | *r* = 0.13, *p* = 0.668 | *r* = -0.25, *p* = 0.394 | *r* = 0.002, *p* = 0.994 |
| ALFF (0.027 ~ 0. 073 Hz) | STG/MTG.L | *r* = -0.17, *p* = 0.566 | *r* = 0.08, *p* = 0.789 | *r* = 0.10, *p* = 0.740 | ***r* = 0.64, *p* = 0.014** | *r* = 0.094, *p* = 0.749 |
|  | CUN/LING/CAL | ***r* = -0.68, *p* = 0.007** | *r* = 0.32, *p* = 0.269 | *r* = -0.20, *p* = 0.484 | *r* = 0.43, *p* = 0.122 | **r = 0.54, p = 0.047** |
|  | IOG.L | ***r* = -0.65, *p* = 0.012** | *r* = 0.16, *p* = 0.595 | *r* = -0.09, *p* = 0.772 | ***r* = 0.70, *p* = 0.005** | *r* = 0.38, *p* = 0.175 |
|  | FFG.R | *r* = -0.48, *p* = 0.081 | *r* = 0.19, *p* = 0.508 | *r* = -0.10, *p* = 0.732 | *r* = 0.35, *p* = 0.217 | *r* = 0.33, *p* = 0.254 |
|  | STG/MTG.R | *r* = -0.37, *p* = 0.195 | *r* = 0.14, *p* = 0.639 | *r* = -0.12, *p* = 0.672 | ***r* = 0.65, *p* = 0.012** | *r* = 0.29, *p* = 0.322 |
| fALFF (0.027 ~ 0. 073 Hz) | MTG.L | *r* = -0.44, *p* = 0.120 | *r* = 0.05, *p* = 0.875 | *r* = -0.12, *p* = 0.670 | ***r* = 0.54, *p* = 0.047** | *r* = 0.24, *p* = 0.408 |
|  | STG.L | *r* = 0.10, *p* = 0.725 | *r* = -0.15, *p* = 0.617 | *r* = -0.41, *p* = 0.150 | *r* = -0.27, *p* = 0.350 | *r* = -0.15, *p* = 0.607 |
| Degree centrality | PostCG.L | *r* = -0.13, *p* = 0.654 | *r* = 0.50, *p* = 0.068 | *r* = -0.11, *p* = 0.717 | ***r* = 0.71, *p* = 0.004** | *r* = 0.44, *p* = 0.113 |
|  | PreCG.L | *r* = 0.08, *p* = 0.778 | *r* = 0.33, *p* = 0.247 | *r* = -0.03, *p* = 0.921 | *r* = 0.45, *p* = 0.110 | *r* = 0.26, *p* = 0.366 |
|  | PostCG.R | *r* = -0.14, *p* = 0.636 | *r* = 0.10, *p* = 0.731 | *r* = 0.20, *p* = 0.503 | *r* = 0.53, *p* = 0.053 | *r* =-0.15, *p* = 0.610 |
|  | Thalamus | *r* = 0.18, *p* = 0.547 | *r* = -0.24, *p* = 0.407 | *r* = -0.19, *p* = 0.523 | ***r* = -0.62, *p* = 0.019** | *r* =-0.08, *p* = 0.776 |
|  | PCC | ***r* = -0.75, *p* = 0.002** | *r* = 0.28, *p* = 0.339 | *r* = 0.15, *p* = 0.603 | *r* = -0.07, *p* = 0.823 | *r* =-0.02, *p* = 0.952 |
|  | Cb.L | *r* = 0.39, *p* = 0.171 | *r* = -0.21, *p* = 0.462 | *r* = 0.26, *p* = 0.371 | ***r* = -0.74, *p* = 0.002** | *r* = 0.22, *p* = 0.458 |
|  | CAU/PUT.R | *r* = 0.25, *p* = 0.398 | *r* = -0.52, *p* = 0.056 | *r* = 0.06, *p* = 0.847 | *r* = -0.52, *p* = 0.059 | *r* = 0.34, *p* = 0.236 |
| Regional homogeneity | CUN/LING/CAL | *r* = -0.58, *p* = 0.029 | *r* = 0.33, *p* = 0.242 | *r* = 0.25, *p* = 0.386 | *r* = 0.37, *p* = 0.195 | *r* =0.48, *p* = 0.079 |

Abbreviations: ALFF, amplitude of low frequency fluctuations; fALFF, fractional amplitude of low frequency fluctuations; PVT, psychomotor vigilance test; KSS, Karolinska Sleepiness Scale; L, left hemisphere; R, right hemisphere; STG/MTG, superior/middle temporal gyrus; CUN/LING/CAL, cuneus/lingual/calcarine; Cb, cerebellum; IOG, inferior occipital gyrus; FFG, fusiform gyrus; PostCG, postcentral gyrus; PreCG, precentral gyrus; PCC, posterior cingulate cortex; CAU/PUT, caudate/putamen.

**Supplementary Table 2** Correlations between the difference (52 hours of sleep deprivation - 14 hours of recovery sleep) in Rs-fMRI metrics and neuropsychological tests for 14 healthy participants.

| A_1_AR distribution volume | | PVT-speed | PVT-lapses | 3-back hits | KSS sleepiness scores | 3-back reaction time |
| --- | --- | --- | --- | --- | --- | --- |
| ALFF (0.01 ~ 0.08 Hz) | MTG/STG.L | *r* = 0.25, *p* = 0.438 | *r* = -0.49, *p* = 0.107 | ***r* = 0.60, *p* = 0.040** | *r* = 0.38, *p* = 0.218 | *r* = -0.34, *p* = 0.277 |
|  | CUN/LING/CAL | *r* = 0.29, *p* = 0.360 | ***r* = -0.72, *p* = 0.008** | ***r* = 0.73, *p* = 0.007** | *r* = -0.28, *p* = 0.371 | *r* = -0.56, *p* = 0.059 |
|  | Cb.R | *r* = -0.35, *p* = 0.260 | *r* = 0.29, *p* = 0.363 | *r* = -0.51, *p* = 0.090 | *r* = 0.009, *p* = 0.979 | *r* = 0.18, *p* = 0.566 |
| fALFF (0.01 ~ 0.08 Hz) | MTG/STG.L | *r* = 0.37, *p* = 0.234 | *r* = -0.50, *p* = 0.098 | *r* = 0.39, *p* = 0.212 | ***r* = -0.59, *p* = 0.044** | *r* = -0.51, *p* = 0.088 |
| ALFF (0.027 ~ 0.073 Hz) | MTG/STG.L | *r* = 0.23, *p* = 0.469 | *r* = -0.49, *p* = 0.109 | ***r* = 0.62, *p* = 0.030** | *r* = -0.42, *p* = 0.174 | *r* = -0.32, *p* = 0.311 |
|  | CUN/LING/CAL | *r* = 0.32, *p* = 0.314 | ***r* = -0.71, *p* = 0.010** | ***r* = 0.72, *p* = 0.009** | *r* = -0.31, *p* = 0.335 | *r* = -0.55, *p* = 0.064 |
|  | IOG.L | *r* = -3.5e-04, *p* = 0.999 | *r* = -0.54, *p* = 0.068 | ***r* = 0.59, *p* = 0.044** | *r* = -0.10, *p* = 0.768 | *r* = -0.31, *p* = 0.333 |
|  | FFG.R | *r* = 0.42, *p* = 0.180 | *r* = -0.60, *p* = 0.041 | *r* = 0.54, *p* = 0.073 | ***r* = -0.59, *p* = 0.046** | *r* = -0.32, *p* = 0.311 |
|  | MTG.R | *r* = 0.14 *p* = 0.675 | *r* = -0.41, *p* = 0.183 | ***r* = 0.69, *p* = 0.013** | *r* = -0.38, *p* = 0.222 | *r* = -0.25, *p* = 0.437 |
| fALFF (0.027 ~ 0.073 Hz) | MTG.L | *r* = 0.31, *p* = 0.320 | *r* = -0.38, *p* = 0.219 | *r* = -0.25, *p* = 0.430 | ***r* = -0.66, *p* = 0.018** | *r* = -0.21, *p* = 0.504 |
|  | STG.L | *r* = 0.29, *p* = 0.356 | *r* = -0.42, *p* = 0.171 | *r* = 0.55, *p* = 0.066 | *r* = -0.57, *p* = 0.051 | *r* = -0.17, *p* = 0.598 |
| Degree centrality | PostCG.L | *r* = 0.34, *p* = 0.285 | ***r* = -0.65, *p* = 0.021** | ***r* = 0.75, *p* = 0.005** | *r* = -0.36, *p* = 0.250 | *r* = -0.42, *p* = 0.169 |
|  | PreCG.L | *r* = 0.40, *p* = 0.198 | ***r* = -0.64, *p* = 0.027** | ***r* = 0.68, *p* = 0.015** | *r* = -0.37, *p* = 0.239 | *r* = -0.46, *p* = 0.129 |
|  | PostCG.R | *r* = 0.37, *p* = 0.243 | *r* = -0.52, *p* = 0.085 | ***r* = 0.68, *p* = 0.015** | *r* = -0.41, *p* = 0.181 | *r* = -0.24, *p* = 0.452 |
|  | Thalamus | *r* = 0.23, *p* = 0.480 | *r* = -0.41, *p* = 0.180 | *r* = 0.58, *p* = 0.050 | *r* = -0.50, *p* = 0.100 | *r* = -0.25, *p* = 0.436 |
|  | PCC | *r* = -0.01, *p* = 0.976 | *r* = -0.27, *p* = 0.394 | ***r* = 0.78, *p* = 0.003** | *r* = -0.18, *p* = 0.572 | *r* = -0.15, *p* = 0.650 |
|  | CAU/PUT.R | *r* = 0.33, *p* = 0.291 | ***r* = -0.69, *p* = 0.014** | ***r* = 0.69, *p* = 0.013** | *r* = -0.33, *p* = 0.296 | *r* = -0.55, *p* = 0.067 |
| Regional homogeneity | CUN/LING/CAL | *r* = 0.38, *p* = 0.229 | *r* = -0.56, *p* = 0.056 | *r* = 0.48, *p* = 0.110 | ***r* = -0.64, *p* = 0.026** | *r* = -0.51, *p* = 0.088 |

Abbreviations: ALFF, amplitude of low frequency fluctuations; fALFF, fractional amplitude of low frequency fluctuations; PVT, psychomotor vigilance test; KSS, Karolinska Sleepiness Scale; L, left hemisphere; R, right hemisphere; STG/MTG, superior/middle temporal gyrus; CUN/LING/CAL, cuneus/lingual/calcarine; Cb, cerebellum; IOG, inferior occipital gyrus; FFG, fusiform gyrus; PostCG, postcentral gyrus; PreCG, precentral gyrus; PCC, posterior cingulate cortex; CAU/PUT, caudate/putamen.
